# On Abelian and Related Fuzzy Subsets of Groupoids

**DOI:** 10.1155/2013/476057

**Published:** 2013-10-29

**Authors:** Seung Joon Shin, Hee Sik Kim, J. Neggers

**Affiliations:** ^1^Department of Physics, University of Michigan, Ann Arbor, MI 48109, USA; ^2^Department of Mathematics, Research Institute for Natural Sciences, Hanyang University, Seoul 133-791, Republic of Korea; ^3^Department of Mathematics, University of Alabama, Tuscaloosa, AL 35487-0350, USA

## Abstract

We introduce the notion of abelian fuzzy subsets on a groupoid, and we observe a variety of consequences which follow. New notions include, among others, diagonal symmetric relations, several types of quasi orders, convex sets, and fuzzy centers, some of whose properties are also investigated.

## 1. Introduction 

The notion of a fuzzy subset of a set was introduced by Zadeh [1]. His seminal paper in 1965 has opened up new insights and applications in a wide range of scientific fields. Rosenfeld [2] used the notion of a fuzzy subset to set down corner stone papers in several areas of mathematics. Mordeson and Malik [3] published a remarkable book, *Fuzzy commutative algebra*, presented a fuzzy ideal theory of commutative rings, and applied the results to the solution of fuzzy intersection equations. The book included all the important work that has been done on *L*-subspaces of a vector space and on *L*-subfields of a field. 

Kim and Neggers [4] introduced the notion of Bin(*X*) and obtained a semigroup structure. Fayoumi [5] introduced the notion of the center *Z*Bin(*X*) in the semigroup Bin(*X*) of all binary systems on a set *X* and showed that a groupoid (*X*, •) ∈ *Z*Bin(*X*) if and only if it is a locally zero groupoid. 

In this paper, we introduce the notion of abelian fuzzy subgroupoids on a groupoid and discuss diagonal symmetric relations, convex sets, and fuzzy centers on Bin(*X*). 

## 2. Preliminaries 

Given a nonempty set *X*, we let Bin(*X*) denote the collection of all groupoids (*X*, ∗), where ∗ : *X* × *X* → *X* is a map and ∗(*x*, *y*) is written in the usual product form. Given elements (*X*, ∗) and (*X*, •) of Bin(*X*), define a product “□” on these groupoids as follows:

(1) $$\begin{array}{c} \left(X,*\right)□\;\left(X,•\right)=\left(X,□\right), \end{array}$$

where

(2) $$\begin{array}{c} x\;□\;y=\left(x*y\right)•\left(y*x\right) \end{array}$$

for any *x*, *y* ∈ *X*. Using that notion, Kim and Neggers proved the following theorem.

**Theorem 1 (see [4])**

(*Bin*(*X*), □) is a semigroup; that is, the operation “□” as defined in general is associative. Furthermore, the left-zero-semigroup is the identity for this operation.

Let *Z*Bin(*X*) denote the collection of elements (*X*, •) of Bin(*X*) such that (*X*, ∗) □ (*X*, •) = (*X*, •) □ (*X*, ∗), for all (*X*, ∗) ∈ Bin(*X*); that is, *Z*Bin(*X*) = {(*X*, •) ∈ Bin(*X*) | (*X*, ∗) □ (*X*, •) = (*X*, •) □ (*X*, ∗), for all (*X*, ∗) ∈ Bin(*X*)}. We call *Z*Bin(*X*) the * center* of the semigroup Bin(*X*).

**Proposition 2 (see [5])**

If (*X*, •) ∈ *Z* *Bin*(*X*), then *x*•*x* = *x* for all *x* ∈ *X*.

**Proposition 3 (see [5])**

Let (*X*, •) ∈ *Z* *Bin*(*X*). If *x* ≠ *y* in *X*, then ({*x*, *y*}, •) is either a left-zero-semigroup or a right-zero-semigroup.

## 3. Abelian Fuzzy Groupoids 

Let (*X*, ∗) ∈ Bin(*X*). A map *μ* : *X* → [0,1] is said to be * abelian fuzzy* if *μ*(*x*∗*y*) = *μ*(*y*∗*x*) for all *x*, *y* ∈ *X*.

**Example 4**

Let (*X*, ∗) be a left-zero-semigroup; that is, *x*∗*y* = *x* for all *x*, *y* ∈ *X*. Let *μ* : *X* → [0,1] be an abelian fuzzy subset of *X*. Then, *μ*(*x*) = *μ*(*x*∗*y*) = *μ*(*y*∗*x*) = *μ*(*y*) for all *x*, *y* ∈ *X*. It follows that *μ* is a constant map.

Similarly, every abelian fuzzy subset of a right-zero-semigroup is also a constant function.

**Proposition 5**

If (*X*, ∗) ∈ *Z* *Bin*(*X*), then every abelian fuzzy subset on (*X*, ∗) is a constant function.

**Proof**

Assume that *μ* is an abelian fuzzy subset of (*X*, ∗). Then, *μ*(*x*∗*y*) = *μ*(*y*∗*x*) for all *x*, *y* ∈ *X*. Let (*X*, ∗) ∈ *Z*Bin(*X*). By Proposition 3, if *x* ≠ *y* in *X*, then ({*x*, *y*}, ∗) is either a left-zero-semigroup or a right-zero-semigroup. It follows that either *x*∗*y* = *x*, *y*∗*x* = *y* or *x*∗*y* = *y*, *y*∗*x* = *x*. In either cases, we obtain *μ*(*x*) = *μ*(*y*) for all *x*, *y* ∈ *X*, proving that *μ* is a constant function.

Given a groupoid (*X*, ∗) ∈ Bin(*X*), we denote the set of all abelian fuzzy subgroupoids on (*X*, ∗) by *A*(*X*, ∗).

**Proposition 6**

Let (*X*, ∗) ∈ *Bin*(*X*). Then, (*X*, ∗) is commutative if and only if *A*(*X*, ∗) = [0,1]^*X*^.

**Proof**

Assume that (*X*, ∗) is not commutative; that is, there exist *x*, *y* ∈ *X* such that *x*∗*y*  ≠*y*∗*x*. If we let *α* : = *x*∗*y* and let *μ* : = *χ*_*α*_ be the characteristic function of *α*, then *μ*(*x*∗*y*) = 1, *μ*(*y*∗*x*) = 0, proving that *μ* is not an abelian fuzzy subset of (*X*, ∗). The converse is straightforward.

Given (*X*, ∗) ∈ Bin(*X*), we define a fuzzy subset *μ*_*α*_ : *X* → [0,1] by *μ*_*α*_(*x*): = *α* for all *x* ∈ *X*, where *α* ∈ [0,1]. Denote by *C*(*X*, ∗): = {*μ*_*α*_ | *α* ∈ [0,1]}. Then, *C*(*X*, ∗)⊆*A*(*X*, ∗) for all groupoids (*X*, ∗) whatsoever. Thus, the extreme of non-commutativity is the situation *C*(*X*, ∗) = *A*(*X*, ∗).

**Proposition 7**

Let *μ* ∈ *A*(*X*, ∗). If *μ* is one-one, then (*X*, ∗) is commutative.

**Proof**

If *μ* ∈ *A*(*X*, ∗), then *μ*(*x*  ∗  *y*) = *μ*(*y*∗ *x*) for all *x*, *y* ∈ *X*. Since *μ* is one-one, we have *x*∗*y* = *y*∗*x* for all *x*, *y* ∈ *X*.

Given (*X*, ∗) ∈ Bin(*X*), we define a set (*X*, ∗)_*a*_ : = {(*x*, *y*) ∈ *X* × *X* | *x*∗*y* = *y*∗*x*}. Note that (*x*, *y*)∈(*X*, ∗)_*a*_ implies (*y*, *x*)∈(*X*, ∗)_*a*_ as well. If we let *ν* : = *χ*_(*X*,∗)_*a*__ be the characteristic function of (*X*, ∗)_*a*_, then *ν* is an abelian fuzzy subgroupoid on *X* × *X*.

**Proposition 8**

Let *μ* : *X* → [0,1] be a fuzzy subset of *X*. If we define (*X*, ∗)_*a*_^*μ*^ : = {(*x*, *y*) ∈ *X* × *X* | *μ*(*x*∗*y*) = *μ*(*y*∗*x*)}, then

- (*X*, ∗)_*a*_⊆(*X*, ∗)_*a*_^*μ*^,
- if *μ* is one-one, then (*X*, ∗)_*a*_ = (*X*, ∗)_*a*_^*μ*^,
- if *μ* is constant, then (*X*, ∗)_*a*_^*μ*^ = *X* × *X*.

**Proof**

It is straightforward.

**Theorem 9**

If (*X*, ∗) ∈ *Bin*(*X*), then there exists a fuzzy subset *μ* of *X* such that (*X*, ∗)_*a*_ = (*X*, ∗)_*a*_^*μ*^.

**Proof**

Assume that there exists (*X*, ∗) ∈ Bin(*X*) such that (*X*, ∗)_*a*_ ≠ (*X*, ∗)_*a*_^*μ*^ for any fuzzy subset *μ* of *X*. Then, there exists an element (*x*, *y*)∈(*X*, ∗)_*a*_^*μ*^∖(*X*, ∗)_*a*_. It follows that *μ*(*x*∗*y*) = *μ*(*y*∗*x*), but *x*∗*y* ≠ *y*∗*x* for some *x*, *y* ∈ *X*. If we let *α*∶ = *x*∗*y* and let *μ*∶ = *χ*_*α*_ be the characteristic function of *α*, then *μ*(*x*∗*y*) = 1, but *μ*(*y*∗*x*) = 0, which proves that (*x*, *y*)∉(*X*, ∗)_*a*_^*μ*^.

**Proposition 10**

Let (*X*, ∗) ∈ *Bin*(*X*) and let · be the usual product on the set of real numbers. If *μ* : (*X*, ∗)→([0,1], ·) is a homomorphism, then *μ* ∈ *A*(*X*, ∗) and (*X*, ∗)_*a*_^*μ*^ = *X* × *X*.

**Proof**

If *μ* : (*X*, ∗)→([0,1], ·) is a homomorphism, then *μ*(*x*∗*y*) = *μ*(*x*) · *μ*(*y*) = *μ*(*y*) · *μ*(*x*) = *μ*(*y*∗*x*) for all *x*, *y* ∈ *X*; that is, *μ* ∈ *A*(*X*, ∗).

If (*x*, *y*) ∈ *X* × *X*, then *μ*(*x*∗*y*) = *μ*(*x*) · *μ*(*y*) = *μ*(*y*∗*x*), proving that (*x*, *y*)∈(*X*, ∗)_*a*_^*μ*^.

**Example 11**

Let (*X*, ∗) ∈ Bin(*X*). Define a binary operation “⋆” on [0, *∞*) by *x*⋆*y* : = (1/2)(*x* + *y*) for all *x*, *y* ∈ [0, *∞*). If *μ* : (*X*, ∗)→([0, *∞*), ⋆) is a homomorphism, then *μ*(*x*  ∗  *y*) = *μ*(*x*)⋆*μ*(*y*) = (1/2)(*x* + *y*) = *μ*(*y*∗*x*) for all *x*, *y* ∈ *X*, which proves that *μ* ∈ *A*(*X*, ∗) and (*X*, ∗)_*a*_^*μ*^ = *X* × *X*.

## 4. Diagonal Symmetric Relations 

Given (*X*, ∗) ∈ Bin(*X*), we denote △(*X*): = {(*x*, *x*) | *x* ∈ *X*}. Then, △(*X*)⊆(*X*, ∗)_*a*_. In particular, if (*X*, ∗) is a left-zero-semigroup, then △(*X*) = (*X*, ∗)_*a*_. 

Let *X*∶ = **R** be the set of all real numbers, and let “−” be the usual subtraction on *X*. Then, (*X*, −)_*a*_ = {(*x*, *y*) | *x* − *y* = *y* − *x*} = {(*x*, *y*) | *x* = *y*} = △(*X*). 

Let *X*∶ = **R** be the set of all real numbers, and let (*X*, ∗, *f*) be a leftoid; that is, *x*∗*y*∶ = *f*(*x*) for all *x*, *y* ∈ *X*, where *f* : **R** → **R** is an even function. If we denote △′(*X*)∶ = {(*x*, −*x*) | *x* ∈ *X*}, then *x*∗(−*x*) = *f*(*x*) = *f*(−*x*) = −*x*∗*x* for all *x* ∈ *X*. It follows that △′(*X*)⊆(*X*, ∗, *f*). 

Let *X* be a nonempty set, and let *S*⊆*X* × *X* such that △(*X*)⊆*S*. *S* is said to be * diagonal symmetric* if (*x*, *y*) ∈ *S*, then (*y*, *x*) ∈ *S* as well. If (*X*, ∗) ∈ Bin(*X*), then (*X*, ∗)_*a*_ is diagonal symmetric. Define a map Φ_*a*_ : Bin(*X*) → *P*(*X* × *X*) by Φ_*a*_(*X*, ∗)∶ = (*X*, ∗)_*a*_.

**Proposition 12**

If *S* is a diagonal symmetric relation on *X*, then there exists (*X*, ∗) ∈ *Bin*(*X*) such that Φ_*a*_(*X*, ∗) = *S*.

**Proof**

Let *S* be a diagonal symmetric relation on *X*. Define a binary operation “∗” on *X* by

(3) $$\begin{array}{c} x*y∶=\{\begin{array}{cc} x & \text{if}\;\;\left(x,y\right)\notin S,\\ z & \text{otherwise}, \end{array} \end{array}$$

where *z* is an element of *X* satisfying *z* = *x*∗*y* = *y*∗*x*. Then, (*X*, ∗)_*a*_ = *S*. In fact, if (*x*, *y*) ∈ *S*, then *x*∗*y* = *y*∗*x*, and hence, (*x*, *y*)∈(*X*, ∗)_*a*_. Assume that (*x*, *y*)∈(*X*, ∗)_*a*_∖*S*. Then, since *S* is symmetric diagonal, we have *x*∗*y* = *x* and (*y*, *x*) ∉ *S*. It follows that *x*∗*y* = *x*, *y*∗*x* = *y*. Since (*x*, *y*)∈(*X*, ∗)_*a*_, we have *x* = *x*∗*y* = *y*∗*x* = *y*, proving that (*x*, *y*) = (*x*, *x*)∈△(*X*)⊆*S*, a contradiction. Hence, Φ_*a*_(*X*, ∗) = (*X*, ∗)_*a*_ = *S*.

If *S* and *T* are diagonal symmetric relations on *X*, then the same is true for *S*∩*T* and for *S* ∪ *T*, while △(*X*) itself is also a diagonal symmetric relation on *X*. In the latter case, the left-zero-semigroup is among the possible groupoids for which Φ_*a*_(*X*, ∗) = (*X*, ∗)_*a*_ = △(*X*).

**Proposition 13**

Let (*X*, ∗), (*X*, •) ∈ *Bin*(*X*). If (*X*, □) = (*X*, ∗) □ (*X*, •), then (*X*, ∗)_*a*_⊆(*X*, □)_*a*_.

**Proof**

If (*x*, *y*)∈(*X*, ∗)_*a*_, then *x*∗*y* = *y*∗*x*, and hence, *x* □ *y* = (*x*∗*y*)•(*y*∗*x*) = (*y*∗*x*) □ (*x*∗*y*) = *y* □ *x*. Hence, (*x*, *y*)∈(*X*, □)_*a*_.

Thus, if (*X*, ∗) and (*X*, □) are given and the question comes up whether (*X*, □) = (*X*, ∗) □ (*X*, •) for some (*X*, •), then (*X*, ∗)_*a*_⊆(*X*, □)_*a*_ is a necessary precondition for this to be the case. For example, if △(*X*) = (*X*, □)_*a*_, then (*X*, ∗)_*a*_ = △(*X*) as well. Thus, we find that in (Bin(*X*), □), “the product does not decrease commutativity” as a general principle.

**Proposition 14**

Let (*X*, ∗) ∈ *Bin*(*X*). If (*A*, ∗) is a subgroupoid of (*X*, ∗), then (*X*, ∗)_*a*_∩(*A* × *A*) = (*A*, ∗)_*a*_.

**Proof**

The proof is straightforward.

**Proposition 15**

Let (*X*, ∗), (*X*, •) ∈ *Bin*(*X*). If (*X*, □) = (*X*, ∗) □ (*X*, •), then *A*(*X*, •)⊆*A*(*X*, □).

**Proof**

If *μ* ∈ *A*(*X*, •), then *μ*(*x*•*y*) = *μ*(*y*•*x*) for all *x*, *y* ∈ *X*. It follows that

(4) μ(x □ y)=μ((x∗y)•(y∗x))=μ((y∗x)•(x∗y))=μ(y □ x).

Hence, *μ* ∈ *A*(*X*, □).

**Proposition 16**

Let (*X*, ∗) ∈ *Bin*(*X*) satisfy the condition: for any *a*, *b* ∈ *X*, there exist *x*, *y* ∈ *X* such that *a* = *x*∗*y*, *b* = *y*∗*x*. If (*X*, □) = (*X*, ∗) □ (*X*, •), then *A*(*X*, •) = *A*(*X*, □).

**Proof**

If *μ* ∈ *A*(*X*, □), then *μ*(*x* □ *y*) = *μ*(*y* □ *x*) for all *x*, *y* ∈ *X*. Given *a*, *b* ∈ *X*, by assumption, we have *x*, *y* ∈ *X* such that *a* = *x*∗*y*, *b* = *y*∗*x*. It follows that

(5) μ(a•b)=μ((x∗y)•(y∗x))=μ(x □ y)=μ(y □ x)=μ((y∗x)•(x∗y))=μ(b•a).

Hence, *μ* ∈ *A*(*X*, •); that is, *A*(*X*, □)⊆*A*(*X*, •). By Proposition 15, we prove the proposition.

Let (*X*, ∗), (*X*, •) ∈ Bin(*X*). Define a relation “≤” on Bin(*X*) by (*X*, ∗)≤(*X*, •) if and only if (*X*, ∗)_*a*_^*μ*^⊆(*X*, •)_*a*_^*μ*^ for any fuzzy subset *μ* : *X* → [0,1].

**Proposition 17**

The relation ≤ is a quasi order on *Bin*(*X*).

**Proof**

Since (*X*, ∗)_*a*_^*μ*^⊆(*X*, ∗)_*a*_^*μ*^ for any fuzzy subset *μ* : *X* → [0,1], we have (*X*, ∗)≤(*X*, ∗) for all (*X*, ∗) ∈ Bin(*X*).

If (*X*, ∗)≤(*X*, •) and (*X*, •)≤(*X*, ⋆), then (*X*, ∗)_*a*_^*μ*^⊆(*X*, •)_*a*_^*μ*^ and (*X*, •)_*a*_^*μ*^⊆(*X*, ⋆)_*a*_^*μ*^, and hence, (*X*, ∗)_*a*_^*μ*^⊆(*X*, ⋆)_*a*_^*μ*^, for any fuzzy subset *μ* : *X* → [0,1]. It follows that (*X*, ∗)≤(*X*, ⋆).

Note that the relation ≤ described in Proposition 17 need not be a partial order on Bin(*X*), since (*X*, ∗)_*a*_^*μ*^ = (*X*, •)_*a*_^*μ*^ for any fuzzy subset *μ* : *X* → [0,1] does not imply (*X*, ∗) = (*X*, •). 

Given (*X*, ∗) ∈ Bin(*X*), we define

(6) [(X,∗)]∶={(X,•)∈Bin(X) ∣ (X,∗)aμ  =(X,•)aμ, ∀μ:fuzzy  subset  of  X}.

Let 〈Bin(*X*)〉∶ =   {[(*X*, ∗)] | (*X*, ∗) ∈ Bin(*X*)}. If we define a relation ≤_*q*_ on 〈Bin(*X*)〉 by

(7) $$\begin{array}{c} \left[\left(X,*\right)\right]\leq_{q}\left[\left(X,•\right)\right]\Leftrightarrow \left(X,*\right)\leq \left(X,•\right). \end{array}$$

then it is easy to see that (〈Bin(*X*), ≤_*q*_〉) is a partially ordered set. For partially ordered sets, we refer to [6].

**Proposition 18**

If [(*X*, ∗)] = [(*X*, •)] in 〈*Bin*(*X*)〉, then *A*(*X*, ∗) = *A*(*X*, •).

**Proof**

Assume that [(*X*, ∗)] = [(*X*, •)]. Then, (*X*, ∗)_*a*_^*μ*^ = (*X*, •)_*a*_^*μ*^ for any fuzzy subset *μ* : *X* → [0,1]. It follows that

(8) μ∈A(X,∗)⇔μ(x∗y)=μ(y∗x) ∀x,y∈X⇔(X,∗)aμ=X×X=(X,•)aμ⇔μ(x•y)=μ(y•x) ∀x,y∈X⇔μ∈A(X,•),

proving that *A*(*X*, ∗) = *A*(*X*, •).

## 5. Convex Sets

**Proposition 19**

Let *μ*, *ν* be fuzzy subsets of (*X*, ∗). If *λ* : = *tμ* + (1 − *t*)*ν*, where *t* ∈ [0,1], then (*X*, ∗)_*a*_^*μ*^∩(*X*, ∗)_*a*_^*ν*^⊆(*X*, ∗)_*a*_^*λ*^.

**Proof**

If (*x*, *y*)∈(*X*, ∗)_*a*_^*μ*^∩(*X*, ∗)_*a*_^*ν*^, then *μ*(*x*∗*y*) = *μ*(*y*∗*x*) and *ν*(*x*∗*y*) = *ν*(*y*∗*x*) for all *x*, *y* ∈ *X*. It follows that

(9) λ(x∗y)=tμ(x∗y)+(1−t)ν(x∗y)=tμ(y∗x)+(1−t)ν(y∗x)=λ(y∗x),

proving that (*x*, *y*)∈(*X*, ∗)_*a*_^*λ*^.

In Proposition 19, the equality does not hold in general. See the following example.

**Example 20**

Let *X* : = [0,1]. Define a binary operation “∗” on *X* by *x*∗*y*∶ = *x* for all *x*, *y* ∈ *X*; that is, (*X*, ∗) is a left-zero-semigroup. Define two fuzzy subsets *μ*, *ν* on *X* by *μ*(*x*)∶ = *x*, *ν*(*x*)∶ = 1 − *x* for all *x* ∈ *X*. Then, *μ* and *ν* are one-one mappings. Let ∈ be a real number such that 0 < ∈<1/2, and let *u*∶ = (1/2)−∈, *v*∶ = (1/2)+∈. Then, *μ*(*v*) − *μ*(*u*) = *ν*(*u*) − *ν*(*v*) ≠ 0. Let *λ*∶ = (1/2)(*μ* + *ν*). Then,

(10) λ(u∗v)−λ(v∗u)  =12[μ(u∗v)+ν(u∗v)]   −12[μ(v∗u)+ν(v∗u)]  =12[μ(u∗v)−μ(v∗u)]   +12[ν(u∗v)−ν(v∗u)]  =12[μ(u)−μ(v)]   +12[ν(u)−ν(v)]=0,

which shows that (*u*, *v*)∈(*X*, ∗)_*a*_^*λ*^.

Since (*X*, ∗) is a left-zero-semigroup and *μ* is one-one, we have (*X*, ∗)_*a*_^*μ*^ = {(*x*, *y*) | *μ*(*x*∗*y*) = *μ*(*y*∗*x*)} = {(*x*, *y*) | *μ*(*x*) = *μ*(*y*)} = △(*X*) = (*X*, ∗)_*a*_^*ν*^. This shows that (*u*, *v*)∉△(*X*) = (*X*, ∗)_*a*_^*μ*^∩(*X*, ∗)_*a*_^*ν*^.

**Corollary 21**

Let *μ*, *ν* be fuzzy subsets of (*X*, ∗). If *λ*∶ = *tμ* + (1 − *t*)*ν*, where *t* ∈ [0,1], and if (*X*, ∗)_*a*_^*μ*^ = (*X*, ∗)_*a*_^*ν*^, then (*X*, ∗)_*a*_^*μ*^ = (*X*, ∗)_*a*_^*ν*^⊆(*X*, ∗)_*a*_^*λ*^.

**Theorem 22**

Let *μ*, *ν* be fuzzy subsets of (*X*, ∗). If *λ*_*i*_∶ = *t*_*i*_ *μ* + (1 − *t*_*i*_)*ν*, (*i* = 1,2), where *t*_1_, *t*_2_ ∈ [0,1] with *t*_1_ ≠ *t*_2_, then (*X*, ∗)_*a*_^*λ*_1_^∩(*X*, ∗)_*a*_^*λ*_2_^⊆(*X*, ∗)_*a*_^*μ*^∩(*X*, ∗)_*a*_^*ν*^.

**Proof**

If (*x*, *y*)∈(*X*, ∗)_*a*_^*λ*_1_^∩(*X*, ∗)_*a*_^*λ*_2_^, then

(11) $$\begin{array}{c} \lambda_{i}\left(x*y\right)-\lambda_{i}\left(y*x\right)=0, \end{array}$$

where *i* = 1,2. It follows that

(12) t1[μ(x∗y)−μ(y∗x)]+(1−t1)  ×[ν(x∗y)−ν(y∗x)]=0t2[μ(x∗y)−μ(y∗x)]+(1−t2)  ×[ν(x∗y)−ν(y∗x)]=0.

Since $\left|\begin{array}{cc} t_{1} & 1-t_{1}\\ t_{2} & 1-t_{2} \end{array}\right|=t_{1}(1-t_{2})-t_{2}(1-t_{1})=t_{1}-t_{2}\neq 0$, by Cramer's rule, we have *μ*(*x*∗*y*) − *μ*(*y*∗*x*) = *ν*(*x*∗*y*) − *ν*(*y*∗*x*) = 0, so that (*x*, *y*)∈(*X*, ∗)_*a*_^*μ*^∩(*X*, ∗)_*a*_^*ν*^. This proves the theorem.

**Corollary 23**

Let *μ*, *ν* be fuzzy subsets of (*X*, ∗), and let *λ*_*i*_∶ = *t*_*i*_ *μ* + (1 − *t*_*i*_)*ν*, (*i* = 1,2), where *t*_1_, *t*_2_ ∈ [0,1] with *t*_1_ ≠ *t*_2_. If (*X*, ∗)_*a*_^*μ*^ = (*X*, ∗)_*a*_^*ν*^, then (*X*, ∗)_*a*_^*λ*_1_^∩(*X*, ∗)_*a*_^*λ*_2_^ = (*X*, ∗)_*a*_^*μ*^ = (*X*, ∗)_*a*_^*ν*^.

**Proof**

It follows immediately from Corollary 21 and Theorem 22.

We give a pause to find some examples of fuzzy subsets *μ*, *ν* of *X* and groupoids (*X*, ∗) and (*X*, •) such that

(13) $$\begin{array}{c} \left(\text{I}\right)\;\;\;\;{\left(X,•\right)}_{a}^{\mu }\subseteq {\left(X,*\right)}_{a}^{\mu },\;\;{\left(X,*\right)}^{\mu }\subseteq {\left(X,•\right)}_{a}^{\nu } \end{array}$$

or

(14) $$\begin{array}{c} \left(\text{II}\right)\;\;\;\;{\left(X,*\right)}_{a}^{\nu }\subseteq {\left(X,*\right)}_{a}^{\mu },\;\;{\left(X,•\right)}^{\mu }\subseteq {\left(X,•\right)}_{a}^{\nu } \end{array}$$

or both (I) and (II) simultaneously.

**Example 24**

Let *X* : = {*a*, *b*, *c*} with the tables:

(15) $$\begin{array}{c} \begin{array}{cccc} {}* & a & b & c\\ a & a & b & c\\ b & c & a & b\\ c & b & c & a \end{array}\;\;\begin{array}{cccc} • & a & b & c\\ a & a & b & c\\ b & b & b & c\\ c & c & c & c \end{array} \end{array}$$

Define two fuzzy subsets *μ*, *ν* on *X* by *μ*(*a*) = 1, *μ*(*b*) = 1/2, *μ*(*c*) = 1/3 and *ν*(*a*) = 1, *ν*(*b*) = *ν*(*c*) = 1/2. Then, it is easy to see that (*X*, ∗)_*a*_ = {(*a*, *a*), (*b*, *b*), (*c*, *c*)} = (*X*, ∗)_*a*_^*μ*^, (*X*, ∗)_*a*_^*ν*^ = (*X*, •)_*a*_^*ν*^ = (*X*, •)_*a*_^*ν*^ = *X* × *X*. Hence, (*X*, ∗)_*a*_^*μ*^⊆(*X*, •)_*a*_^*μ*^, (*X*, •)_*a*_^*ν*^ = (*X*, ∗)_*a*_^*ν*^, and (*X*, ∗)_*a*_^*μ*^⊆(*X*, ∗)_*a*_^*ν*^, (*X*, ∗)_*a*_^*ν*^⊆(*X*, •)_*a*_^*ν*^.

## 6. Fuzzy Center 

Let (*X*, ∗) ∈ Bin(*X*), and let *μ* be fuzzy subsets of *X*. Define a set *Z*_*μ*_(*X*, ∗) by

(16) $$\begin{array}{c} Z_{\mu }\left(X,*\right)∶=\left\{x\in X\;|\;\mu \left(x*y\right)=\mu \left(y*x\right)\right\}. \end{array}$$

We call it a *μ-center* of (*X*, ∗). In Example 24, *Z*_*μ*_(*X*, ∗) = *∅*, *Z*_*ν*_(*X*, ∗) = *X*.

**Proposition 25**

Let (*X*, ∗) ∈ *Bin*(*X*). Then, *x* ∈ *Z*_*μ*_(*X*, ∗) if and only if ({*x*} × *X*)∪(*X* × {*x*})⊆(*X*, ∗)_*a*_^*μ*^.

**Proof**

It follows that

(17) x∈Zμ(X,∗)⇔μ(x∗y)=μ(y∗x), ∀y∈X⇔(x,y),(y,x)∈(X,∗)aμ⇔({x}×X)∪(X×{x})⊆(X,∗)aμ.

Let (*X*, ∗) ∈ Bin(*X*), and let *μ* : *X* → [0,1] be a fuzzy subset of *X*. Define a set *OZ*_*μ*_(*X*, ∗) by

(18) OZμ(X,∗)∶={x∈X  μ(x∗y)  =μ(y∗x)⇒μ(x)=μ(y)}.

With the notion of *μ*-center, we obtain the following.

**Proposition 26**

If *OZ*_*μ*_(*X*, ∗)∩*Z*_*μ*_(*X*, ∗) ≠ *∅*, then *μ* is a constant function.

**Proof**

If *x* ∈ *OZ*_*μ*_(*X*, ∗)∩*Z*_*μ*_(*X*, ∗), then *μ*(*x*∗*y*) = *μ*(*y*∗*x*) for all *y* ∈ *X*. Since *x* ∈ *OZ*_*μ*_(*X*, ∗), we have *μ*(*x*) = *μ*(*y*) for all *y* ∈ *X*, proving that *μ* is a constant function.

**Proposition 27**

If *μ* is a constant function, then *OZ*_*μ*_(*X*, ∗) = *X* = *Z*_*μ*_(*X*, ∗).

**Proof**

*μ* is a constant function, then *μ*(*x*∗*y*) = *μ*(*y*∗*x*) for all *x*, *y* ∈ *X*. It follows that *Z*_*μ*_(*X*, ∗) = *X*.

Assume that *x* ∉ *OZ*_*μ*_(*X*, ∗). Then, there exists an *y* ∈ *X* such that *μ*(*x*∗*y*) = *μ*(*y*∗*x*), but *μ*(*x*) ≠ *μ*(*y*). Since *μ* is a constant function, we obtain *μ*(*x*) = *μ*(*y*) ≠ *μ*(*x*), a contradiction. Hence, *OZ*_*μ*_(*X*, ∗) = *X*.
